# Adaptive Sampling for Urban Air Quality through Participatory Sensing

**DOI:** 10.3390/s17112531

**Published:** 2017-11-03

**Authors:** Yuanyuan Zeng, Kai Xiang

**Affiliations:** 1 Electronic Information School, Wuhan University, Wuhan 430072, China; 2 Collaborative Innovation Center for Geospatial Technology, Wuhan 430079, China; 3 School of Information Management and Statistics, Hubei University of Economics, Wuhan 430205, China

**Keywords:** urban sensing, air quality sensing, data sampling, adaptiveness

## Abstract

Air pollution is one of the major problems of the modern world. The popularization and powerful functions of smartphone applications enable people to participate in urban sensing to better know about the air problems surrounding them. Data sampling is one of the most important problems that affect the sensing performance. In this paper, we propose an Adaptive Sampling Scheme for Urban Air Quality (AS-air) through participatory sensing. Firstly, we propose to find the pattern rules of air quality according to the historical data contributed by participants based on Apriori algorithm. Based on it, we predict the on-line air quality and use it to accelerate the learning process to choose and adapt the sampling parameter based on *Q*-learning. The evaluation results show that AS-air provides an energy-efficient sampling strategy, which is adaptive toward the varied outside air environment with good sampling efficiency.

## 1. Introduction

The Urban Air Quality pollution issue becomes the main prominent social problem all over the world nowadays with the high-speed development of economy. When considering the fast growth of urbanization and fossil fuel consumptions, our country is facing the greatest challenge that has never been encountered before. According to the Environmental Performance Index Report 2016, published by Yale University [1], China is still the most serious district of PM2.5 pollution. The government is now strengthening the policy of air quality pollution prevention. For the expensive building cost of air monitoring stations and limited coverage of stations, the air quality pollution prevention is still a big challenge. Beyond this, urban air quality is also affected by many factors, including meteorology and human factors leading to more difficulties. On the other hand, the public pays more attention to the environment problems surrounded. Other than to achieve the air quality information through the government channel, they prefer fine-grained ambient air quality information related with their health issues in an active and timely way. According to the data from the biggest on-line shopping website in China, there was shopping records with millions of PM2.5 prevention masks being sold in only one day. More and more people are also willing to pay money for portable PM2.5 detection devices that can be carried around. The above shows that people have the willingness to participate in urban air quality sensing and make contributions to the urban environment.

The popularization of the smartphone enables people to participate in urban sensing with kinds of build-in sensors for the smartphone or through the sensor resources connected to the phone plug-in or wirelessly. To some degree, people can be viewed as human sensors that join in the urban sensing to provide their feelings toward the urban air. Our work is different from the official air quality monitoring. More than to provide general PM2.5 measurement, participatory urban air sensing aims at human-aware and fine-grained air environment monitoring, especially for a destined location area and time duration. In urban air participatory sensing, we encourage people join in sensing tasks and be aware of the ambient air environment. It may not be the exactly accurate measurement, but can provide human-centric air quality information that can be used as a guide for air environment protection. Participatory sensing can integrate urban sensing and human activity together, and then helps to improve the sensing coverage and efficiency. The sensing process will also be merged with social resources that help with information processing and sharing.

In urban air quality participatory sensing, data sampling is one of the key problems to sensing performance. The sampling strategy needs to match the air quality situations effectively with modest energy consumptions of the smartphone. According to the census data, air pollution is tightly connected with human activities [2]. For example, the air pollution data in Baolian station, Haidian District of Beijing, from 2009 to 2012 [3] shows PM2.5 and CO air pollutant is with lower density in weekend than weekday. It is so called “weekend effect”. Beyond this, air quality also shows “holiday effect”. PM2.5, CO and SO_2_ pollutant shows an unusual higher level than non-holiday days in specific holidays. For example, during Spring Festivals, air quality was worse than the usual days. It may be related with the festival fireworks burning in Spring Festival. Fixed data sampling cannot satisfy the various sensing environment. Intuitionally, a high sampling frequency may be wasteful if lacks interesting air quality events, such as in good air situations. Low sampling frequency may not guarantee efficient measurement toward the air quality event, such as in serious air pollution. Beyond this, participants try to save energy to ensure the necessary daily communications of smartphone. One of the solutions is to adapt the data sampling strategy as needed. In this paper, we propose an Adaptive Sampling Scheme for Urban Air Quality (AS-air) through participatory sensing, which aims to choose an energy-efficient data sampling parameter that is adaptive to the varied air environment.

The main research contribution of this paper includes:

1. It has been found that urban air shows some specific patterns such as the “weekend effect” and the “holiday effect”. By knowing the pattern rules, it helps to decide the sampling parameter. In AS-air, we firstly design the method to find the pattern rules between sensing data and urban air quality by digging the correlations between them based on Apriori algorithm.
2. Based on it, we then propose an adaptive sampling scheme by using *Q*-learning to adapt sampling parameter according to the outside sensing environment. According to the learned pattern rules, we predict the on-line air quality during sensing and use it to accelerate the *Q*-learning process of sampling strategy to convergence.
3. As-air provides an energy-efficient sampling scheme that is adaptive to the outside air environment with good sampling performance, which is suitable for mobile and on-and-off application scenarios for smartphone participatory sensing.

The following of the paper is organized as follows. Section 2 presents the related work. Section 3 presents the system overview. We present the details of AS-air in Section 4. Section 5 is performance evaluation. Finally, Section 6 concludes this paper.

## 2. Related Work

In the past few decades, Wireless Sensor Networks (WSNs) got incredible attentions as one of the effective solutions for environmental sensing. But wireless sensor networks are constrained by deployment and devices. Nowadays, the human-centric smart system [4] arouses more interests that can integrate ubiquitous sensing data from the physical and cyber space to discover a certain pattern connected with our daily life. Estrin et al. [5] propose the concept of participatory sensing, which is the process whereby individuals and communities use evermore-capable mobile phones and cloud services to collect and analyze systematic data for use in revealing a certain pattern of the city. A series of systems and frameworks are presented afterwards.

### 2.1. System Frameworks for Participatory Sensing

Common Sensor [6] proposed by UC Berkley is a mobile sensing system that is based on handheld devices, which allows for the public and community to join in the air quality measurement. Rana et al. [7] propose Ear-phone, which is an open participant sensing platform implemented on Nokia N95 and HP iPAQ for urban noise detection. Hasenfratz et al. [8] introduce a participatory air quality mobile sensing system, GasMobile, which is based on small, low-cost, and off-the-shelf hardware to monitor the ozone concentration. Sun et al. [9] propose a deployed and participatory sensing system for urban air quality monitoring, which monitors the environmental parameters through deployed sensor nodes or human-centric sensing that is provided by build-in sensors of smartphone. Tse et al. [10] design and realize a certain personal pollution awareness system tiny enough to be embedded into user accessories, which can be utilized for real-time air quality monitoring.

Another closely connected concept is crowdsensing. Crowdsensing is with a similar paradigm as participatory sensing, while it includes not only participatory sensing way but also opportunistic sensing. See et al. [11] propose that crowdsensing can be used for citizen sensing application scenario, and they find that active contribution (emphasized in participatory sensing especially) is more envisioned than passive contribution with citizens' motivation by the desire to aid a worthy cause with little training. With the participatory governance features, the case study work in crowdsensing for transportation and environmental related urban sensing has been proposed in [12,13,14].

### 2.2. Sampling Data Recruitment in Participatory Sensing

Sampling data acquisition is mostly based on data recruitment in participatory sensing. Three main categories are included according to the metrics in data recruitment.

The first category is coverage-based data recruitment. D. Estrin et al. [15] propose the data recruitment schemes according to the geographical position of participants and time-related task coverage, as well as habits of participants. Hamid et al. [16] propose the data acquisition scheme to recruit the smart vehicles for urban sensing according to the vehicle trajectory by analyzing the tempo-spatial coverage.

The second category is reputation-based data recruitment. In Reference [15], they also propose a reputation-based recruitment by computing the reputation based on Beta distribution. Amintoosi et al. [17] propose reputation-based recruitment framework for social participatory sensing according to the trust mechanism based on friend and friend's friend. In [18], they propose the recruitment by considering both the quality of contribution and the trustworthiness level of participants within the social networks. Alswailim et al. [19] propose a reputation system to evaluate participants by grouping participants based on their contributions and then selecting the highest group value based on their reputation values.

The third category is expertise-based data recruitment. Researchers have done some work on this in social networks [20,21]. In which, expert finding models are proposed that aim at identifying persons with relevant level of knowledge or experience for a given topic. Borges et al. [22] propose a tier-2 participatory urban infrastructure monitoring platform that is based on the level of knowledge and process capabilities of citizens and domain experts (such as municipal authorities).

Actually, most of the current sampling data recruitment design considers more than one factor and combine them together [18,23,24,25,26].

The above recent research work focuses on how to select participants to contribute their data by recruitment in participatory sensing. During the sensing process, the data recruitment method will help to recruit participants that can provide qualified data in consideration of human mobility and budget, etc. Data recruitment solves the problem of where and who to make data sampling. Our work focus on adaptive data sampling strategy that is built based on the existing data recruitment schemes.

As far as we know, there has been no paper published about adaptive sampling especially for participatory sensing yet. The adaptive sampling related algorithms are normally discussed in wireless sensor networks.

### 2.3. Adaptive Sampling in Wireless Sensor Networks

For wireless sensors that have limited power budget, energy-aware data sampling algorithms are proposed in order to reduce the energy consumption of sensors and extend network lifetime. The conventional methods with a fixed sampling rate incur extra activity of the sensor node. Energy-aware adaptive sampling algorithms modify the sampling rate of the sensors according to the needs of sensing tasks [27,28,29]. Srbinovski et al. [30] propose an energy-aware adaptive sampling algorithm for energy harvesting WSN to adapt the sampling frequency according to the energy of nodes. Since WSNs are often application-oriented, the design of adaptive sampling algorithms has to consider these factors. Leonard et al. [31] propose adaptive ocean sampling for autonomous ocean observing and prediction system. They propose a performance metric to derive optimal paths for mobile sensors in order to minimize error in a model estimate of the sampled field. Graham et al. [32] propose a distributed algorithm for adaptive sampling of spatiotemporal processes whose mean is unknown and covariance is known up to a scaling parameter. The optimal sampling strategy aims to minimize the average of the prediction error variance. Xu et al. [33] propose an adaptive sampling strategy based on the learning Gaussian process in order to minimize the information-theoretic cost function of the Fisher Information Matrix. Nguyen et al. [34] propose an information-driven adaptive sampling strategy in mobile robotic wireless sensor networks, which aims to minimize the uncertainty at all of the unobserved locations of interest. Salim et al. [35] propose an adaptive sampling approach for wireless body sensor networks to estimate and adapt the sensing frequency based on previous readings and patient criticality. The adaptive sampling approach aims to handle emergency detection with energy saving. Silva et al. [36] propose adaptive sensing to adapt the sampling frequency in order to capture the behavior of the physical parameters of interest and then reduce the overhead in terms of sensing events.

From the above, we can see that data sampling is a critical problem for participatory sensing. Current research mainly focus on data recruitment schemes, but are lacking efficient and intelligent sampling strategy to support it. Although adaptive sampling has been studied and used in wireless sensor networks, the available solutions are not well suited for participatory sensing. In participatory sensing, the sensing devices are smart handheld devices such as smartphone, which is more capable than general sensors. Beyond this, participatory sensing provides the human-centric sensing scenarios. Human intervention makes the urban air quality demonstrating certain patterns of air quality. Making full use of human-related pattern of urban air for data sampling helps to improve the sensing performance. As far as we know, this is the first time adaptive sampling scheme by considering air pattern in participatory sensing for urban air monitoring has been proposed.

## 3. System Overview

**Figure 1:**
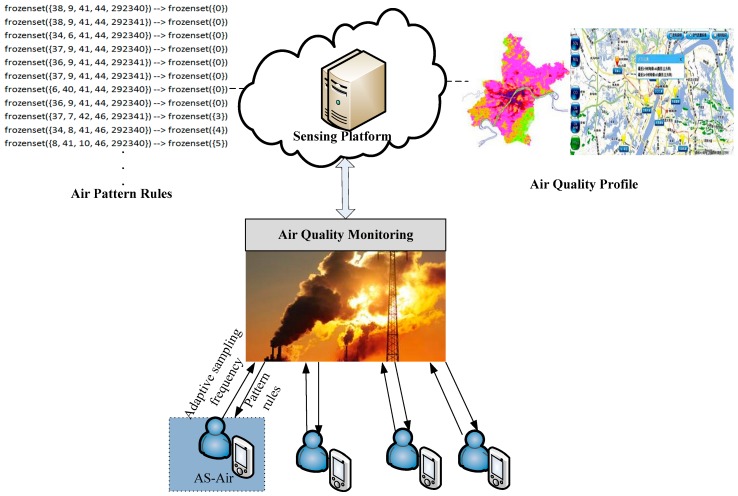
Participatory Sensing System for Urban Air Quality Monitoring.

The general structure of a typical participatory sensing system is illustrated in Figure 1, which consists of a platform usually on the cloud and smartphone participants. In the paper, we involve this general system framework into urban air quality monitoring. It aims to provide personal pollution awareness toward urban air quality. People with portable smart devices, such as smartphones, are invited to participate in urban air quality sensing. With the smartphone built-in sensors and plug-in air detection devices, data are sensed such as GPS, image, temperature, humidity, and PM2.5 concentration, etc. Except that, people with smartphones can also be looked at as human sensors. They can provide their feelings about the current air environment, such as comfortable or uncomfortable. They contribute data sensed by smartphones to the platform with their daily activities. The urban sensing platform will process the contributed data and will finally forman application-specific profile, i.e., air quality profile. Different from the traditional air quality monitoring by fixed locations and numbers of watching stations, participatory sensing based system provides human-aware and fine-grained air quality information by encouraging people to join in the ambient air sensing. During the sensing process, the data recruitment scheme will help to recruitment participants that can provide qualified data with accuracy and timeliness, etc. The discrepancy brought by factors, such as measurement condition and human mobility, can be further processed by using the data fusion algorithm to try to reduce discrepancies and outliers. Our AS-air is an adaptive data sampling strategy that is built upon the recruitment scheme. So, we have the assumptions that qualified participants have been recruited for the current sensing task and even with a valid incentive mechanism to encourage them to contribute sensing data effectively.

We focus on adaptive sampling scheme AS-air in the paper. AS-air is committed on the smartphone of participant side. We propose an adaptive sampling scheme AS-air to dynamically adapt the sampling parameter according to varied outside environment. The varied air situation is analyzed by the sensing platform according to the historical sensing data, and then forms air pattern rules. Each participant acquires the pattern rules from the platform and makes prediction on the future air quality. Based on the predicted air quality, they learn to adapt suitable sampling parameter accordingly.

## 4. Adaptive Sampling Scheme for Urban Air Quality Sensing (AS-Air)

### 4.1. Urban Air Pattern Rules

In this subsection, we explain how to make analysis on the historical sensing data and form air pattern rules. The air pattern implies certain association rules that are related to many factors, such as participant activity and meteorology condition, etc. To find out the pattern rules, we use association rule learning [37] to make data mining based on historical data.

The historical sensing data stored in the sensing platform is formatted into a series of records, including fields such as trajectory and meteorology, e.g., (*participant_id*, *latitude*, *longitude*, *time*, *date*, *temperature*, *humidity*, *weather*, *pollutant concentratin*, *...*).

To discover the pattern rules, the sensing platform makes some preliminaries upon the historical data records. According to the location precision needs of sensing tasks, each location that is represented by *latitude* and *longitude* is divided into grids as *grid_0_*, *grid_2_*…*grid_n_*. The sensing time represented by *time* item is divided into hour slots within a day as *slot*_1_, *slot*_2_,…*slot_m_*, according to sensing needs. According to *date* item, we know sensing happened in weekend or weekday, and the specific season, i.e., spring, summer, autumn and winter. The temperature and humidity represented by *temperature*, *humidity* item is divided into range levels such as: very high, high, average, low, and very low. The weather represented by *weather* item belongs to one of the predefined weather types such as windy, rainy and sunny, etc. The *pollutant concentration* data is with concentration of pollutant that reflects air quality such as *PM*2.5, *PM*10, etc. They are used to calculate Individual Air Quality Index (IAQI), and then the Air Quality Index (AQI) level. So, the sensing data record of participant can be represented as: (*participant_id*, *grid*, *slot*, *week*, *season*, *temp_level*, *hum_level*, *weather*, *AQI_level*).

According to the AQI level ranking standard that is widely used in our country nowadays, there are six levels of AQI: good, moderate, unhealthy for sensitive groups, unhealthy, very unhealthy, and hazardous, respectively. In AS-air, we propose to use Apriori algorithm to process the historical data records of participants and find the rules for a certain AQI level. The pattern rule can be shown as: (*grid*, *slot*, *week*, *season*, *temp_level*, *hum_level*, *weather → AQI level*). In which, the priori in the rule can be only one item or combinations of them. For example, the pattern rules generated as: “frozenset({38, 9, 41, 44, 292340}) --> frozenset({0})” means the specific day of the week, season, temperature, humidity, and location grid can deduce the specific air quality level. In which, the *frozenset* function is the invariable set function in Python. The filed such as *week*, *season*, *temp_level*, and *hum_level* has been represented by numbers within the individual specific range.

### 4.2. Adaptive Sampling Strategies

Data sampling is one of the main energy consumption sources of participants‘ smartphone, for they still need enough power to support the communication functions of their daily usage. Obviously, fixed period of sampling incurs many disadvantages. Fixed periodic sampling ignores the change of air quality events throughout the sensing process. High sampling rate can be wasteful if there are no emergent air quality events. Low sampling rate may not satisfy the sensing needs toward the specific air quality event. A solution to save energy is to use sampling duty cycle by periodically turning on sampling module during sensing. We thus design AS-air that dynamically adapts the sampling parameter according to the air environment. Participatory sensing provides the human-centric sensing scenarios. Human intervention makes the urban air showing certain patterns that affect sensing results. By using *Q*-learning, we try to learn the outside environment information combining multiple factors, such as meteorology and human factors. Different from general *Q*-learning procedure, we use the pattern rules for adaptive sampling by considering the predicted air quality behind the rules that can guide the sampling strategy. It helps to choose and adapt the sampling strategy to fasten the convergence to the optimal strategy, which is well suited for mobile scenarios of smartphone participants.

**Figure 2:**
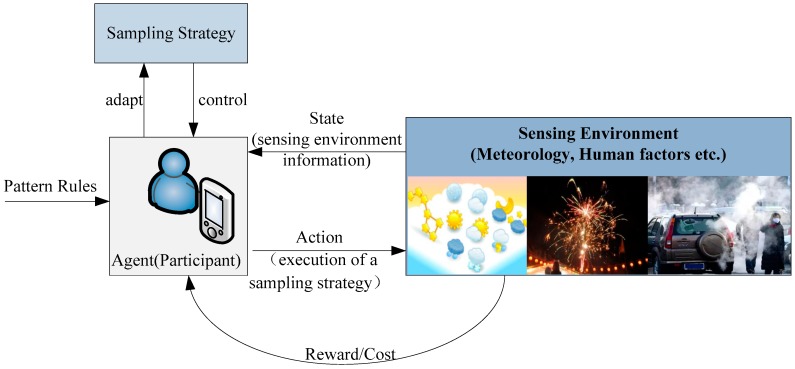
Adaptive Sampling Scheme for Urban Air Quality (AS-air) based on *Q*-learning.

AS-air suggests an intelligent control problem that adapts sampling parameter based on sensing environment and smartphone energy resources. The problem is then formulated by distributed independent reinforcement learning [38]. During the intelligent learning procedure, each participant (corresponds to an agent) adapts its sampling strategy and then converges to the optimal strategy according to the reward/cost with different actions toward the sensing environment. We use *Q*-learning, a form of model-free reinforcement learning to circumvent the adaptive sampling for urban air quality through participatory sensing. *Q*-learning is simple with modest computational resources that is well suited for smartphone applications. By using *Q*-learning, we try to learn the outside sensing environment information such as meteorology, human factors, et al, and use both those factors and predicted air situation by pattern rules to choose and adapt sampling strategy. Figure 2 shows AS-air scheme based on *Q*-learning. Each participant chooses sampling strategy locally according to both the pattern rules and the state of the sensing environment information monitored. The execution of the chosen sampling strategy is an action toward the environment. In reverse, the action will incur a reward/cost back to the participant. The participant will adapt the sampling strategy until the reward/cost converges to the objective with an optimal strategy found.

The participatory sensing system by using reinforcement learning is formulated by (*S*, *A*, *R*, *Ť* ), where *S* is the discrete air quality sensing state space. *A* is the discrete action space that is dependent on sampling strategies taken. *R*: *S* × *A→***R** is the cost function, which implies the quality of a state-action combination of the system. *Ť*: *S* × *A→*Δ*S* is the state transition function, where Δ*S* is the probability distribution over state space *S*. In our AS-air scheme, the actions include sets of sampling parameter strategies of participants. The strategy of sampling parameter is taken by learning the outside sensing environment scenarios. The strategy includes the adaptation of sampling period *t* toward sensing environment accordingly. The sensing time is divided into a non-overlapping equal time period that is called a frame. Each frame is then divided into non-overlapping equal time slots according to sensing time precision. For example, a day is chosen as a frame in urban air quality participatory sensing. Each day is divided into time slots by an hour. We define sampling energy consumption, *E*(*t*), as the amount of sampling energy consumption in each frame, which is calculated in Equation (1). In which, *e_s_* is the energy consumed per sampling. *T* is the duration of a frame.

(1) $$E(t)=e_{s}\frac{T}{t}$$

According to Nyquist theorem, the minimum sampling frequency *f* = 1/*t* should be more than double of the maximum frequency *f_s_* = 1/*t_s_* in the power spectrum of the signal to guarantee the reconstruction of the sampled signal, i.e.,

(2) $$0<t<\frac{1}{2}t_{s}$$

The reward/cost function for each participant is defined as the amount of energy consumptions for the *i*th frame, which is shown as in (3). Then, the energy consumption *Q*-value in the *i*th frame with state *s* that takes action *a* by current strategy is evaluated as in (4). In which, *α* is the learning rate and in the range of (0,1). *γ* is the discount factor and is also in the range of (0,1). We use a constant learning factor, and the learning procedure can track the sensing environment.

(3) $$R(i)=\left\{ \begin{array}{l} E_{i}(t)=e_{s}\frac{T}{t_{i}}\;if\;satisfy\;(2)\\ \infty \;otherwise \end{array}\right.$$

(4) $$Q(s,a)=(1-\alpha )\cdot Q(s,a)+\alpha (R_{i}+\gamma \;\underset{a}{\min}\;Q(s^{\prime },a))$$

During the learning process, AS-Air has to explore all of the possible strategies based on air quality situations, and then choose the “good” strategy. The strategy exploration probability is chosen by Equation (5). In which, *k* is a constant that can be tuned to control the effect of unexplored strategies. The minimum exploration *ε_min_* is required to deal with the dynamic sensing environment. With the heuristic exploration policy, an initial exploration is with a higher rate when the participants join in the air quality sensing. It then gradually decreases over time. *t_ref_* is reference sampling time interval, which is decided according to the predicted AQI level, as described in Section 4.1.

(5) $$\varepsilon =\varepsilon_{\min}+\max(0,k\cdot |t_{ref}-t|/t_{ref})$$

## 5. Performance Evaluation

In this Section, we make performance evaluation of AS-air. The adaptive sampling strategy in AS-air is based on the pattern rules digging from the historical sensing data. Beyond this, AS-air needs dataset to testify the performance. We built our own dataset with the sensing data that was collected through the experimental system developed. In the following, we introduce the experimental system and how we collect the data at first. Next, we make simulations on AS-air to testify to the efficiency and effectiveness.

### 5.1. Experimental System and Dataset Built

Our experimental platform consists of two parts: the air sensing platform and android-based client. The platform server is built based on Spring and Mybatis framework. The client supports Android 4.4 OS and the version above that. The air sensing platform is responsible for sensing task publication, data store, data process, and event result publication. The client includes user registration, air sensing module, localization service, and data service. The air quality monitoring task such as “monitoring air quality in Electronic Information School, Wuhan University” is published through the platform. Participants that install the android-based client software will decide whether to join the task according to their own interest. Participants acquire local air quality related data sensed by smartphone built-in and plug-in sensors such as: GPS and USB plug-in PM2.5 detector, and then deliver the data to the platform server. In the experiment system, we still use fixed sampling parameter in air sensing module. Through the platform, we acquire the historical sensing data through participatory sensing as the dataset and use it for further simulation-based evaluation in subsection B.

The dataset is built upon nine people, four months, five districts spread in Wuhan city area through participatory sensing. Each data record in the dataset includes fields as: (*participant_id*, *time_slot_no*,*location_grid_no*, *day_of_the_week*, *temperature_level*, *humidity_level*, *AQI_level*). Among the data record, the *location_grid_no* field can be acquired through smartphone localization, such as GPS and WiFi based method. The *time_slot_no* and *day_of_the_week* fields can be acquired from smartphone by calculation. The *temperature_level*, *humidity_level* and *AQI_level* fields can be acquired through USB plug-in PM2.5 detector with plantower PMS5003ST. For people have no USB plug-in PM2.5 detector available, they provide their personal feelings about the urban air, i.e., people behave as sensors. Since our system aims to provide peoples’ awareness about the ambient air environment, subjective feeling rate ranking about urban air, but the accurate value is not allowed. In the experiment system, we provide people to upload their feelings by six levels as (comfortable, acceptable, lightly allergic, allergic uncomfortable, very uncomfortable) according to the six AQI levels from 0 to 5 of air quality.

**Figure 3:**
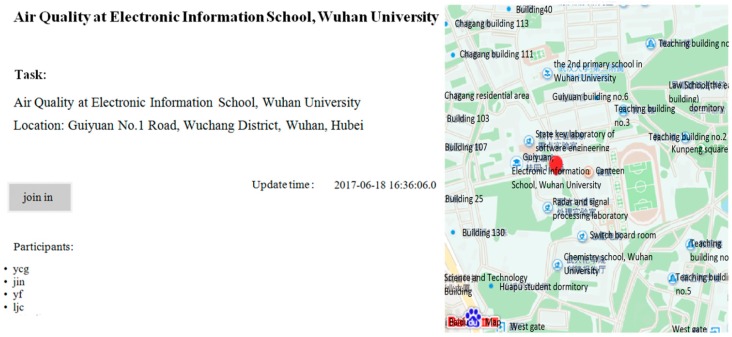
The screenshot of air sensing platform webpage.

**Figure 4:**
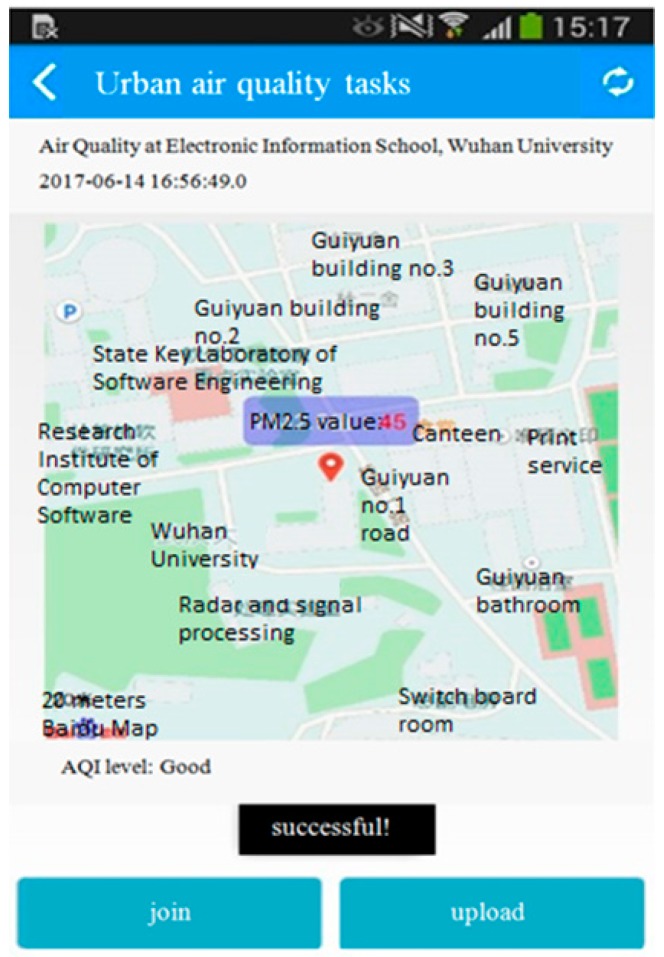
The screenshot of air sensing android-based smartphone client.

**Figure 5:**
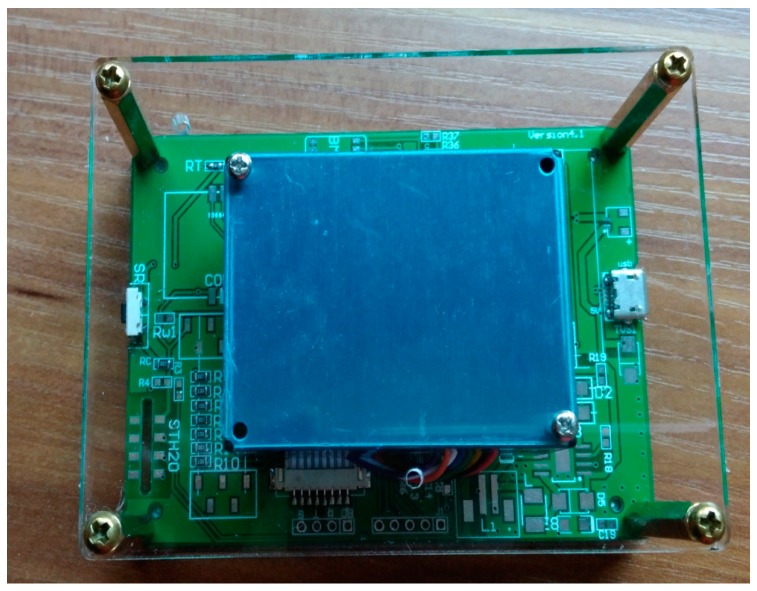
The PM2.5 detector.

The screenshot of server web and client APP is shown as in Figure 3 and Figure 4. Figure 3 shows the air sensing task publication at Electronic Information School, Wuhan University. Figure 4 shows the android-based smartphone participant uploading the PM2.5 data sensed locally. Our system is developed in Chinese version. The key text is translated into English in the screenshot. Figure 5 shows the PM2.5 detector with plantower PMS5003ST we used in our experiment. According to PM2.5 data sensed, we can calculate the PM2.5 AQI (individual AQI) and corresponding AQI level. The detector can be attached to smartphone through mini USB interface.

### 5.2. Simulations of AS-Air

We then evaluate the performance of AS-air by simulations. Our simulation-based evaluations are based on the dataset formed through the experimental system. The adaptive sampling strategy is based on the air pattern. We make evaluations on Apriori-based pattern rules learning at first. The random 50% portion of the dataset is used for Apriori-based pattern rules learning. The others are for adaptive sampling in AS-air.

#### 5.2.1. Apriori-Based Pattern Rules Learning

**Figure 6:**
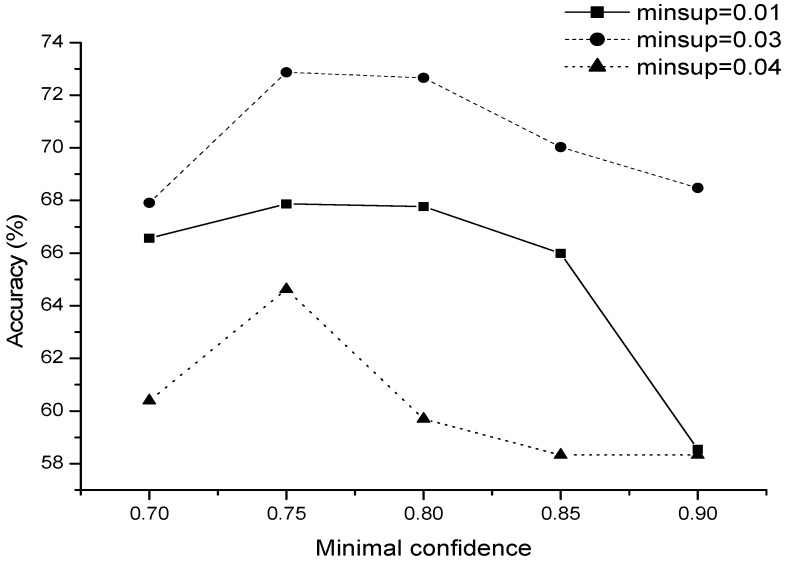
The accuracy of air quality pattern learning.

There are two key parameters in Apriori algorithm: the minimal support ratio and minimal confidence level. The selection of the two parameters affects the results of association rules. Figure 6 shows the accuracy of Apriori-based pattern rules learning with different minimal support and confidence parameters. The results show that we achieve an acceptable prediction performance when choosing suitable minimal support with considerable confidence. When minimal support ratio is set to 0.04, we have the accuracy around and more than 70% with good confidence level (more than 70% confidence level).

#### 5.2.2. AS-Air Evaluations

We mainly consider the following performance metrics in the evaluation: sampling energy ratio, sampling efficiency, and adaptiveness. Sampling energy ratio is defined as the ratio between the total time of a frame and the active sampling time, which is proportional to the sampling energy efficiency. Sampling efficiency is defined as the average qualified percentage of sampling period parameter chosen that matches with the current sensing task. Adaptiveness is defined as the adaptiveness of AS-air according to the varied sensing environment, i.e., to self-adapt the sampling parameter according to the outside sensing environment. We initialize the parameter values as: *α* = 0.5, *γ* = 0.5. Set *k* = 0.25, *ε_min_* = 0.1. The time frame *T* is set to 24 h. We then present the simulation results, respectively.

**Figure 7:**
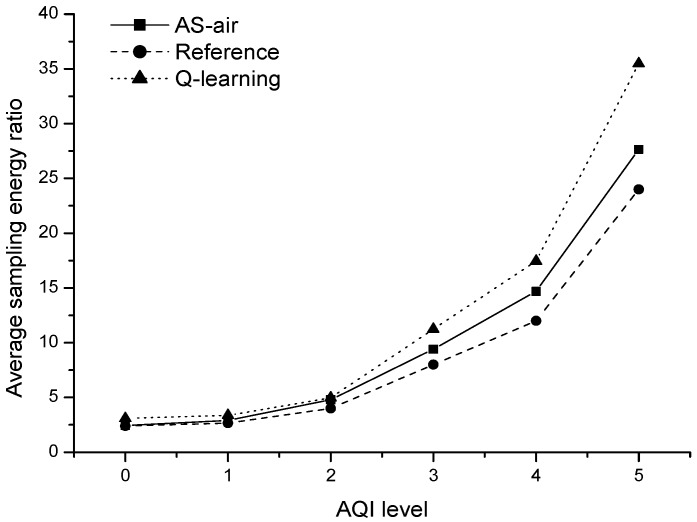
The average sampling energy ratio with different Air Quality Index (AQI) level.

Figure 7 shows the average sampling energy ratio with different AQI level from 0 to 5. As shown in Equation (1), sampling energy ratio is proportional to sampling energy consumptions. From the results, we can see that: the sampling energy consumptions increase gradually as the air quality becomes worse. It is reasonable because worse air quality implying severe air pollution events that need more frequent sampling. In the results, we also make comparisons with reference sampling ratio and *Q*-learning based average sampling ratio. The reference sampling ratio is the needed sampling ratio that matches with the specific AQI level. The reference is decided according to a long-time sensing process. The *Q*-learning based average sampling ratio is achieved through basic *Q*-learning algorithm to optimize the sampling parameter. We can see that: AS-air has better sampling energy efficiency when compared with the basic *Q*-learning based algorithm. AS-air can be well tuned to and approaches the optimized sampling parameter quickly, but *Q*-learning based algorithm cannot. AS-air has a slightly worse sampling energy efficiency when compared with reference. AS-air only predicts the air quality pattern by Apriori algorithm, but cannot know the exact air quality situation. So the efficiency of AS-air is dependent on the accuracy of air quality pattern learning. As shown in Figure 6, the air pattern rules learning in AS-air can provide the acceptable accuracy and confidence when choosing appropriate parameters.

**Figure 8:**
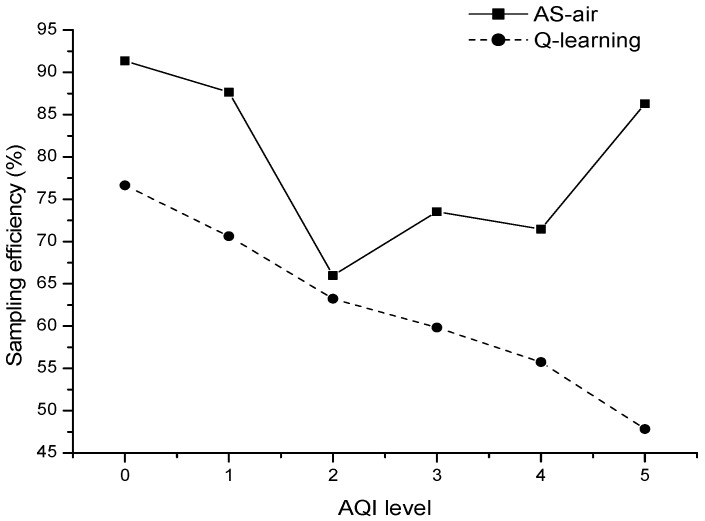
The sampling efficiency with different AQI level.

Figure 8 shows the sampling efficiency with different AQI level from 0 to 5 when we make comparisons between AS-air and basic *Q*-learning based algorithm. The results show that AS-air has much better sampling efficiency within most of the AQI situations. In AS-air, we predict the air quality situation and then adapt the sampling parameter with *Q*-learning based on it. The basic *Q*-learning based method only tunes the sampling parameter by the reward function obtained during each execution of the sampling task. So, AS-air has a better sampling performance (with more qualified sampling strategy toward the sensing environment). It is easy to find the optimized parameter during the learning procedure. The results also show that AS-air has a slightly worse sampling efficiency when the AQI level equals to 2 by comparing with the other AQI levels. This has something to do with the dataset. Seldom data in the dataset focuses on the situations with AQI equals to 2. So, we learn fewer rules from the dataset, and then poor prediction accuracy with it. This then leads to a poor sampling efficiency.

**Figure 9:**
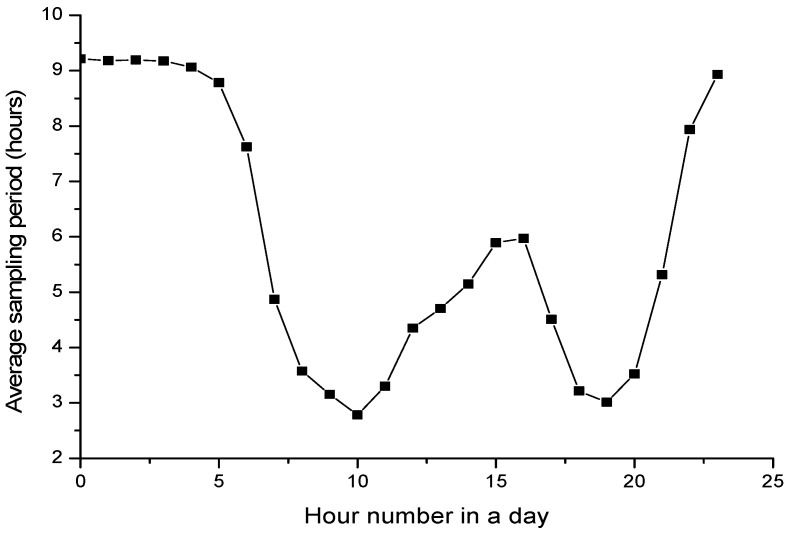
The average sampling period within different hour in a day.

**Figure 10:**
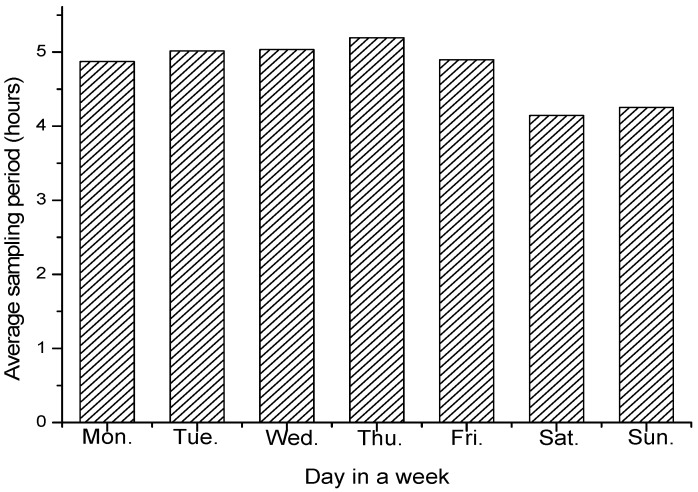
The average sampling period within the day in a week.

**Figure 11:**
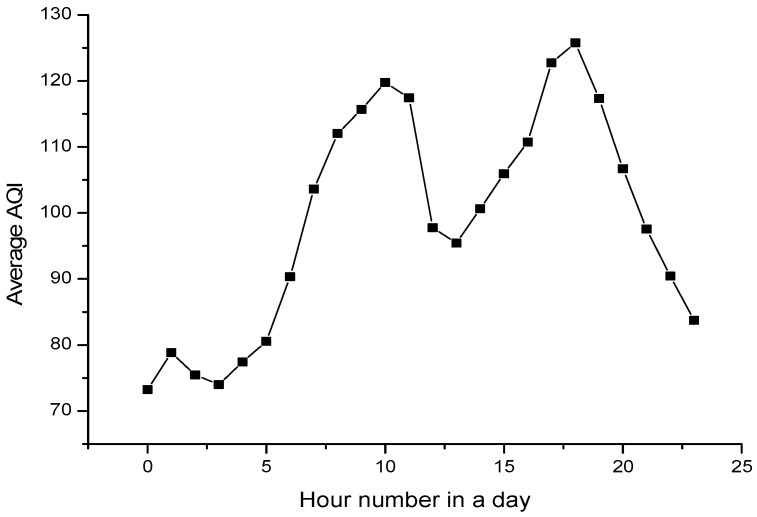
The average AQI within different hour in a day.

**Figure 12:**
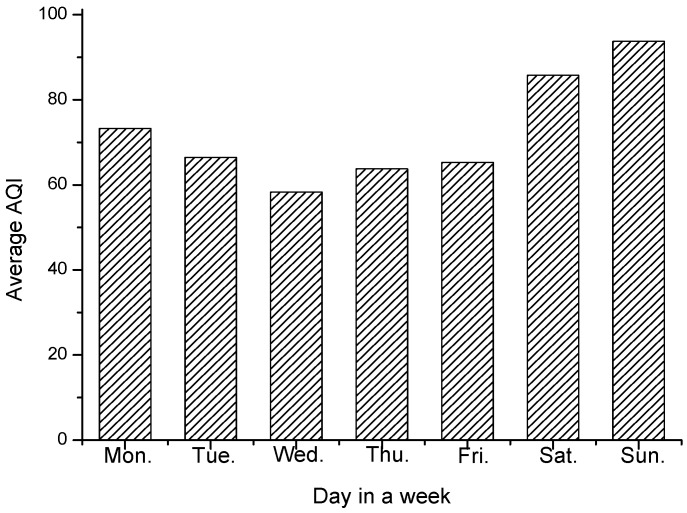
The average AQI within the day in a week.

To evaluate the adaptiveness, we record the change of average sampling parameter within a day and a week to see if it is adaptive toward the air quality situations. Figure 9 and Figure 10 shows the average sampling period with different hour of the day and different day of the week, respectively. Figure 11 and Figure 12 shows the change tendency of average AQI within a day and a week. The results show that AS-air chooses the sampling parameter that is self-adaptive to the air quality situations. When air quality becomes worse, the sampling period will be shortened. When air quality becomes better, the sampling period will be extended. From Figure 9 and Figure 10, we can find out that there are two serious air quality hour zones (around 7 am–10 am, 5 pm–7 pm) in a day, and then the sampling period in AS-air will be adaptive toward this air quality situation change. From Figure 12, we can see that weekend is with much worse air quality when compared with a weekday. In Figure 11, AS-air is with shortened sampling period in weekend, which implies that our scheme is adaptive to the air quality of the day in a week.

In this Section, the true dataset that is collected through the experimental system built is used for simulation-based evaluations of AS-air. The evaluation results of Apriori-based pattern rules learning are with acceptable prediction by choosing suitable algorithm parameters (i.e., minimal support ratio and minimal confidence level). The efficiency of AS-air is dependent on the accuracy of air quality pattern learning. AS-air has better sampling energy efficiency and sampling efficiency (with more qualified sampling strategy) when compared with basic *Q*-learning based algorithm. AS-air can be well tuned to and approaches the optimized sampling parameter quickly, which is suitable for mobile and on-and-off application scenarios for smartphone participatory sensing. The performance AS-air is also related with the accuracy of pattern rules learning. More accurate pattern rules help the learning procedure of the optimized sampling strategy in AS-air. AS-air chooses the sampling parameter that is adaptive to the air environment. When air quality becomes worse, the sampling period will be shortened. When air quality becomes better, the sampling period will be extended.

## 6. Conclusions and Future Work

In this paper, we propose AS-air scheme for urban air quality through participatory sensing, which provides an energy-efficient adaptive sampling scheme toward the varied air environment. AS-air can predict the on-line air quality situation with acceptable accuracy based on the analysis of historical dataset by air pattern rules learning through Apriori algorithm. Based on this, AS-air proposes adaptive sampling strategies based on *Q*-learning. The predicted on-line air quality from air pattern rules help to accelerate the convergence to the optimal sampling strategy. Our evaluation is based on the true dataset that is collected through the experiment system built. The results show that AS-air can choose and adapt suitable sampling parameter that is adaptive toward the urban air environment. AS-air provides sampling strategy with good energy-efficiency, sampling performance, and adaptiveness when compared with basic *Q*-learning based scheme. Our future work includes the implementation and evaluation of AS-air in our experimental system and more people, and a longer time-span data collection will be involved. Another concern is about the quality of sampling data and the potential work on data quality analysis and evaluations [39,40]. We will do some work about this in the future.
